# Supplementary material for: Sub-millisecond conformational dynamics of the A2A adenosine receptor revealed by single-molecule FRET
Source: Commun Biol. 2023 Apr 3;6:362. doi: 10.1038/s42003-023-04727-z (PMC10070357; doi:10.1038/s42003-023-04727-z)
Supplement: Supplementary file 2 — Supplementary Information [file 42003_2023_4727_MOESM2_ESM.pdf]

Supplementary Information

Sub-millisecond conformational dynamics of the A_{2A} adenosine receptor revealed by single-molecule FRET

Maslov et al.

Supplementary figure 1. *Labeling and characterization of the double-cysteine mutant of A_{2A}AR.* 2
Supplementary figure 2. *Schematic presentation of the home-built MFD-PIE confocal setup.* 3
Supplementary figure 3. *Stoichiometry and ALEX-2CDE filters isolate fluorescence bursts corresponding to the double-labeled receptor subpopulation in single-molecule experiments.* 4
Supplementary figure 4. *Simulations show that deviations of bursts from the static FRET line in the E vs. τ_D plot are statistically significant.* 5
Supplementary figure 5. *Simulations show that the FRET-2CDE scores in A_{2A}AR are significantly higher than expected for static molecules.* 6
Supplementary figure 6. *The cumulative distribution of FRET-2CDE scores in double-labeled A_{2A}AR molecules shows increased dynamics for the agonist-bound A_{2A}AR at all threshold levels.* 7
Supplementary figure 7. *Microtime patterns (top) and filter functions (bottom) used for fFCS.* 8
Supplementary figure 8. *FCS in single-labeled A_{2A}AR molecules reveals photoblinking on a microsecond timescale* 9
Supplementary figure 9. *Fitting of experimental data for A_{2A}AR with a fFCS model including one anticorrelation term.* 10
Supplementary figure 10. *PDA histograms for the model with two dynamic states* 11
Supplementary figure 11. *PDA histograms for the model with three static states.* 12
Supplementary figure 12. *PDA histograms for the fFCS-constrained PDA model.* 13
Supplementary figure 13. *Fluorescence depolarization shows moderate reorientation freedom of the dyes attached to A_{2A}AR in apo and all ligand-bound conditions* 14
Supplementary figure 14. *MD simulations suggest that a dye attached to TM6 can enter the G-protein-binding cavity upon A_{2A}AR activation.* 15
Supplementary figure 15. *Inter-label distances calculated from MD simulations.* 16
Supplementary figure 16. *Convergence of MD sampling* 17
Supplementary figure 17. *Distributions of the burst-wise fluorescence lifetime (τ) and anisotropy (r) for fluorophores in (A) donor-only and (B) acceptor-only A_{2A}AR populations.* 21
Supplementary table 1. *Labeling efficiencies* 22
Supplementary table 2. *Mean burst-wise parameters* 23
Supplementary table 3. *Fitting parameters for fFCS cross-correlation curves.* 24
Supplementary table 4. *Fitting parameters for a PDA model with two dynamic states.* 25
Supplementary table 5. *Fitting parameters for a PDA model with three static states.* 26
Supplementary table 6. *Fitting parameters for an fFCS-constrained PDA model.* 27
Supplementary table 7. *Fitting parameters for ensemble-based fluorescence depolarization curves* 28
Supplementary References 28

Supplementary figure 1. Labeling and characterization of the double-cysteine mutant of A2AAR.

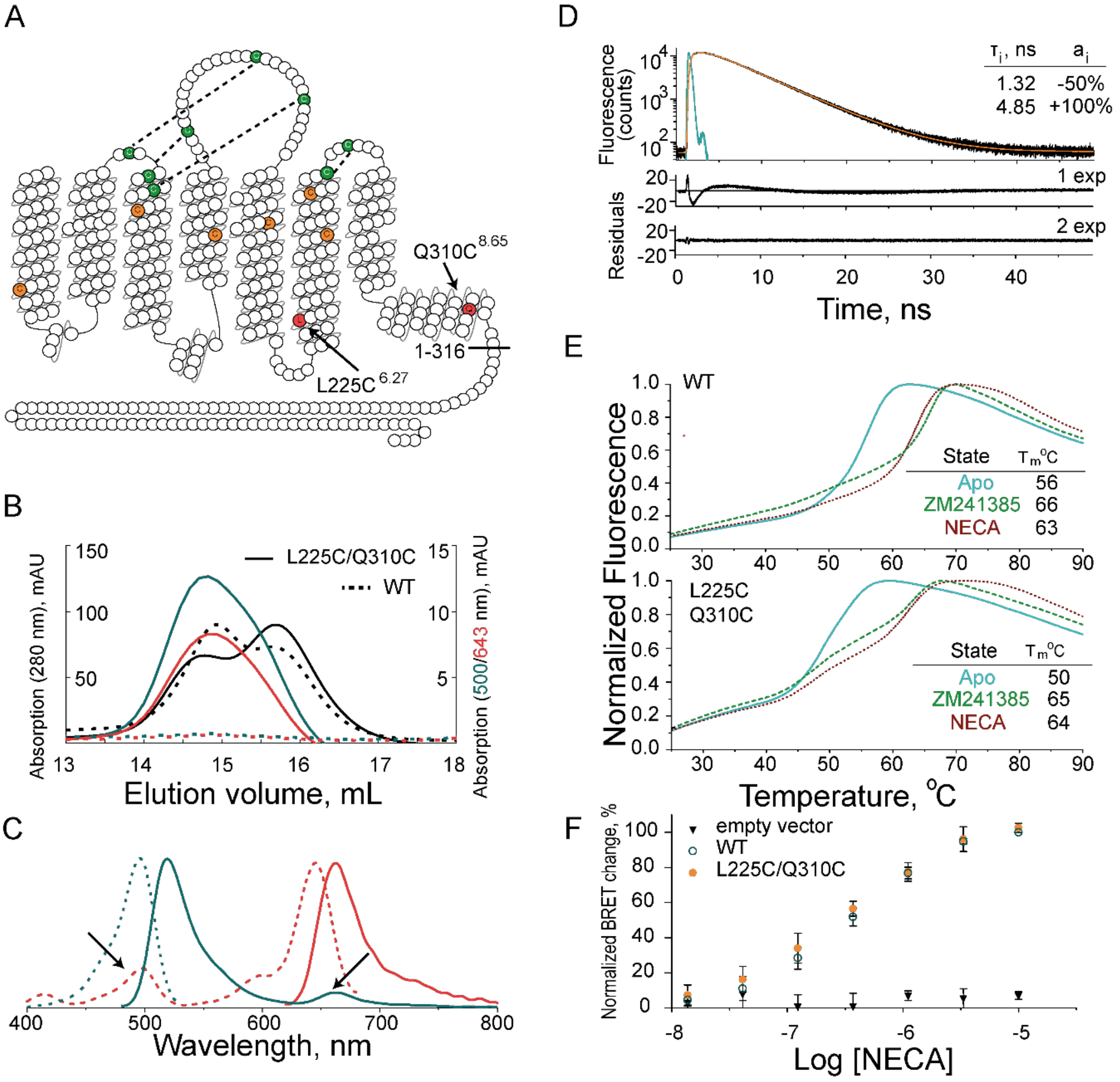

(A) A "snake plot" of the amino acid sequence of A2AAR (adapted from gpcrdb.org) shows the labelling sites (L225C$^{6.27}$ and Q310C$^{8.65}$) for fluorescent dyes (red circles, black arrows). The native Cys-bridges and unpaired cysteines in A2AAR are marked in green and orange, respectively. The C-terminus of A2AAR was truncated at residue 316. **(B)** Size-exclusion chromatography of the wild-type (dashed lines) and mutant (solid lines) apo-A2AAR in lipid nanodiscs. Dark cyan, red, and black lines correspond to absorption at 500, 643, and 280 nm, respectively. The rightward-shifted peak of the absorption at 280 nm corresponds to the scaffold MSP1D1 protein in empty nanodiscs. **(C)** Fluorescence excitation (dashed lines) and emission (solid lines) spectra of double-labeled apo-A2AAR in lipid nanodiscs indicate FRET (peaks shown with arrows). Donor (dashed, dark cyan) and acceptor (dashed, red) excitation spectra were measured with emission fixed at 550 nm and 700 nm, respectively. Donor (solid, dark cyan) and acceptor (solid, red) emission spectra were measured with excitation fixed at 460 nm and 600 nm, respectively. **(D)** The TCSPC pattern of acceptor emission after pulsed donor excitation shows a rising term associated with FRET in the double-labeled A2AAR. Black and orange lines show experimental data and their biexponential fit, respectively. Cyan line shows the IRF (Instrument Response Function) of the setup. Residuals for monoexponential and biexponential fits are shown below the main panel. Parameters of the biexponential fit (fluorescence lifetime and relative amplitudes) are given in the insert table. **(E)** TSA for WT (top frame) and unlabeled double-cysteine mutant (bottom frame) of A2AAR bound to ZM241385 (dashed green line), NECA (dashed red line), or apo-A2AAR (cyan line) in nanodiscs. Melting temperature (T_m) values are given in the insert tables. **(F)** BRET assay shows cAMP signaling induced with agonist NECA in HEK293T cells transfected with a plasmid containing WT (open dark cyan circles) or unlabeled double-cysteine mutant A2AAR (closed orange circles) gene. Black triangles show a lack of ligand-response in the cells transfected with an empty vector. Data points correspond to the mean ± SD of n = 3 biological replicas. In each experiment, the change in BRET efficiency was normalized to the maximum BRET efficiency change in the WT receptor.

Supplementary figure 2. Schematic presentation of the home-built MFD-PIE confocal setup

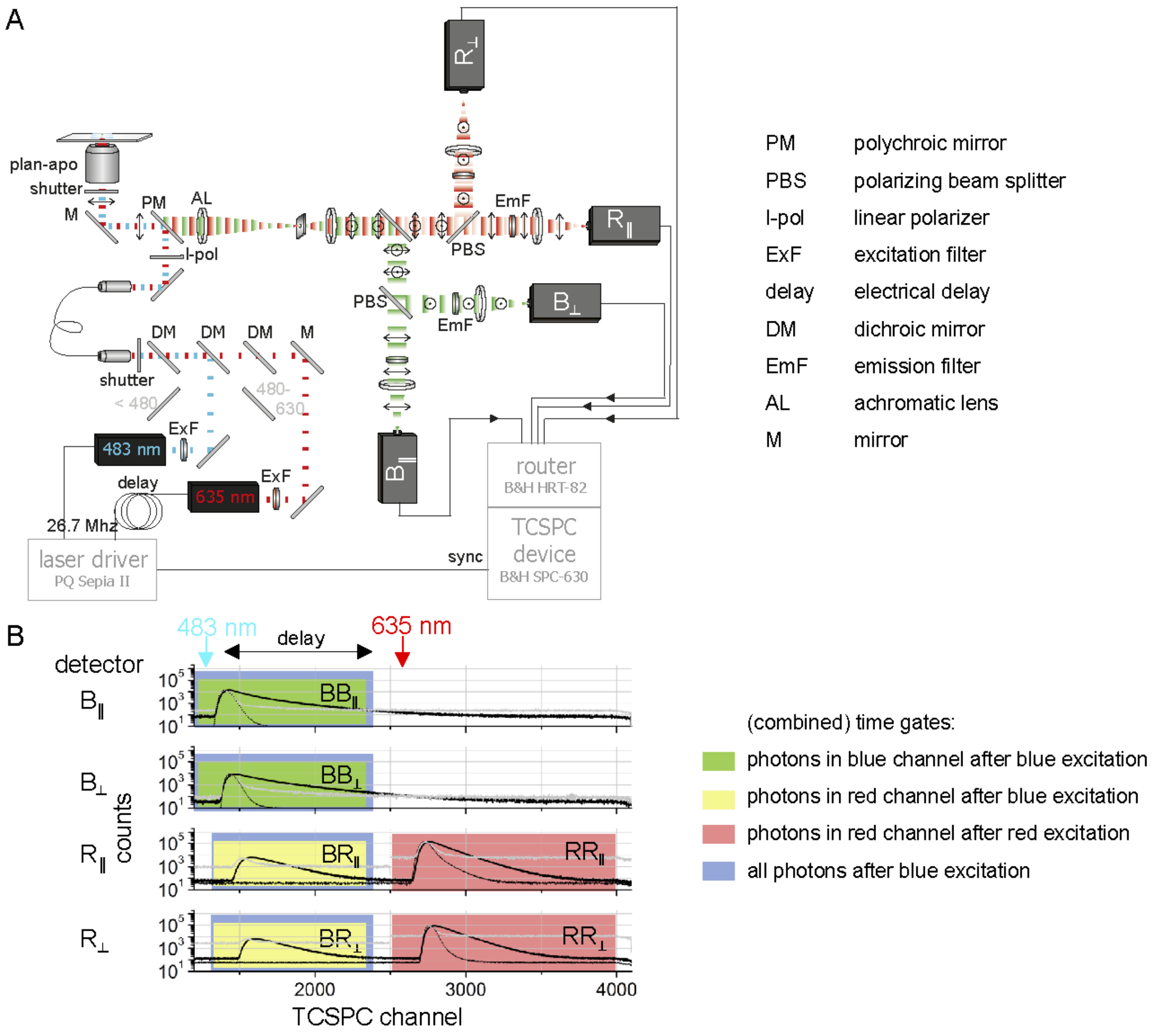

(A) Schematic presentation of the home-built MFD-PIE confocal setup[1]. Two pulsed lasers (483 nm and 635 nm) with a ~18 ns lag time, were combined in a single-mode optical fiber, the linear polarization was cleaned up and reflected into the microscope. The transmitted sample emission was focused through a pinhole and spectrally split. Each color (B - blue, R - red) was separately split in two detection channels according to their polarization. Resulting four channels ($B_{\|}$, $B_{\perp}$, $R_{\|}$, $R_{\perp}$) were detected in TCSPC mode. **(B)** Different time gates ('PIE channels') in the previously described detection channels depending on the blue or red excitation line. Data (thick black line), IRF (thin black line), background recording (gray) are illustrated, along with the nomenclature of the different detectors and time gates. Since the 635 nm laser is delayed, first photons are detected on the $BB_{\|}$/$BB_{\perp}$ (donor direct excitation) and $BR_{\|}$/$BR_{\perp}$ (FRET-channel) time gates. After the delay, 635 nm excitation triggers photons in the $RR_{\|}$ and $RR_{\perp}$ time gates (direct excitation of acceptor).

Supplementary figure 3. Stoichiometry and ALEX-2CDE filters isolate fluorescence bursts corresponding to the double-labeled receptor subpopulation in single-molecule experiments.

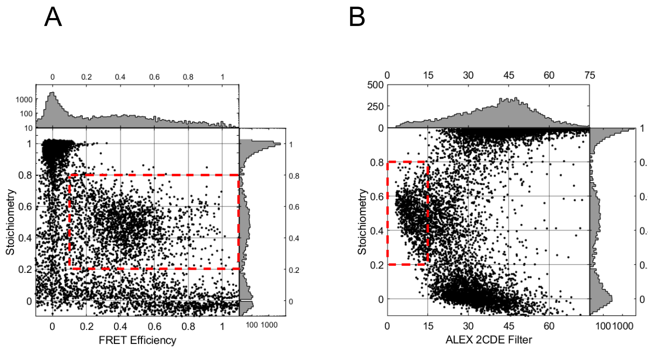

(A) Scatter plot of stoichiometry S against FRET efficiency – the donor-only molecules are centered around the top-left corner, the acceptor-only molecules cluster in the bottom of the plot space. The double-labeled molecules in the red rectangle were selected for the further analysis. **(B)** Scatter plot of stoichiometry *S* against ALEX-2CDE filter value. The donor-only and acceptor-only molecules tend to the top-right and bottom-right parts of the plot, respectively. The double-labeled molecules in the red rectangle were selected for further analysis.

Supplementary figure 4. Simulations show that deviations of bursts from the static FRET line in the E vs. τ_D plot are statistically significant.

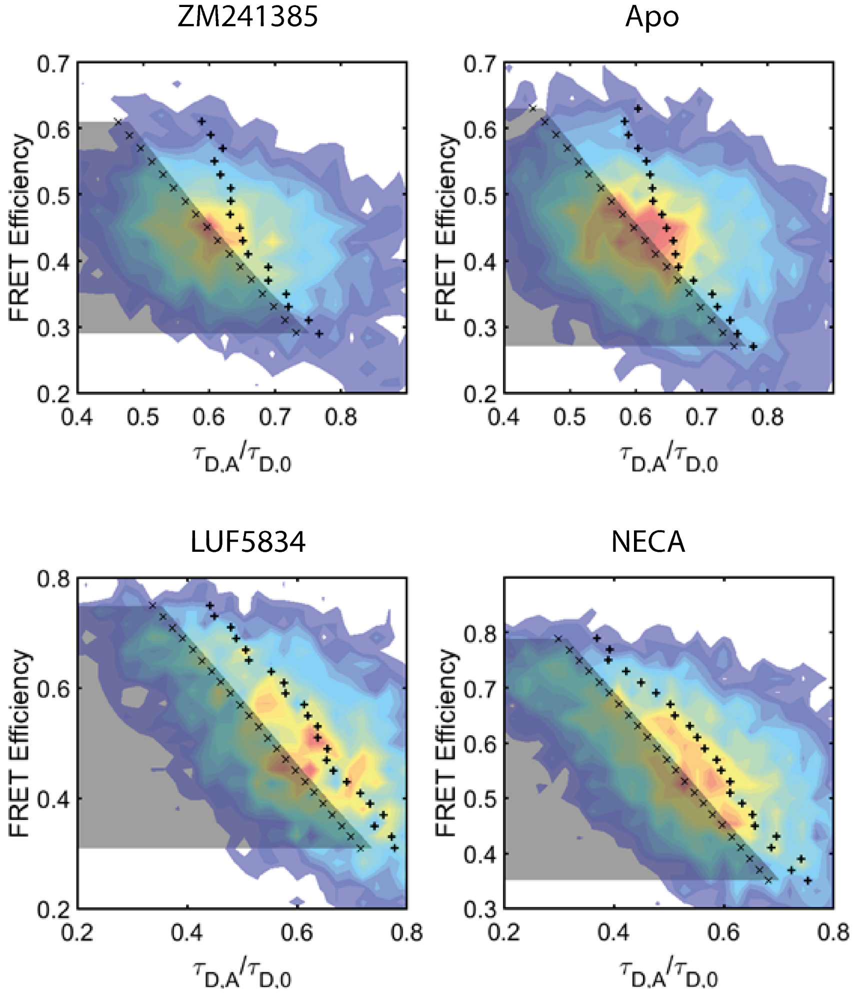

The measured distributions of the double-labeled molecules across burst-wise FRET efficiency and burst-wise donor lifetime are shown for apo and ligand-bound conditions in colored contour plot. All bursts were split in groups of similar FRET efficiency, in each group the weighted donor lifetime was calculated and shown as '+'. The mean weighted donor lifetime simulated for static molecules and the upper limit of the 99.9%-confidence interval are shown as 'x' and filled grey area. For more details, see "Burst-wise fluorescence lifetime".

Supplementary figure 5. Simulations show that the FRET-2CDE scores in $A_{2A}AR$ are significantly higher than expected for static molecules.

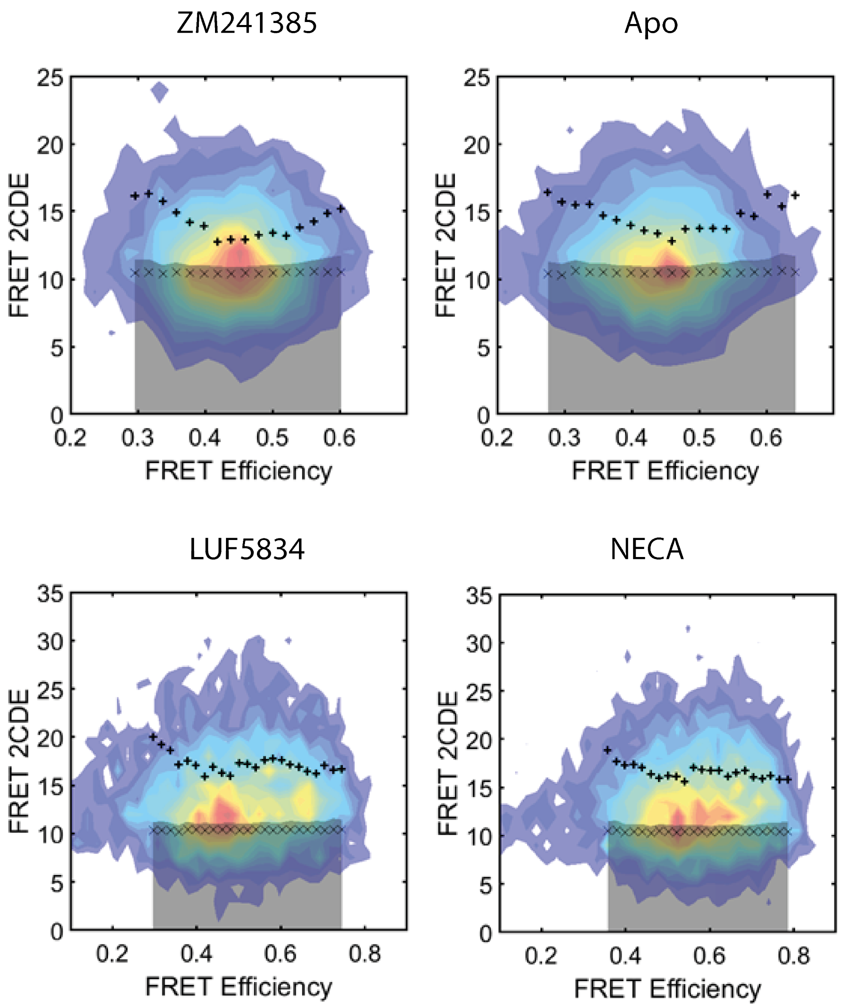

The measured distributions of the double-labeled $A_{2A}AR$ receptors across burst-wise FRET efficiency and the burst-wise FRET-2CDE scores ('+' symbols) are shown for all four apo and ligand-bound conditions in colored contour plot. All bursts were split in groups of similar FRET efficiency, in each group the intensity-weighted FRET-2CDE score was calculated and shown as straight (vertical / horizontal) crosses. The mean intensity-FRET-2CDE scores for simulated static molecules and the upper limit of the 99.9%-confidence interval are shown with crosses ('x') and filled grey area. For more details, see "FRET-2CDE analysis".

Supplementary figure 6. The cumulative distribution of FRET-2CDE scores in double-labeled $A_{2A}AR$ molecules shows increased dynamics for the agonist-bound $A_{2A}AR$ at all threshold levels.

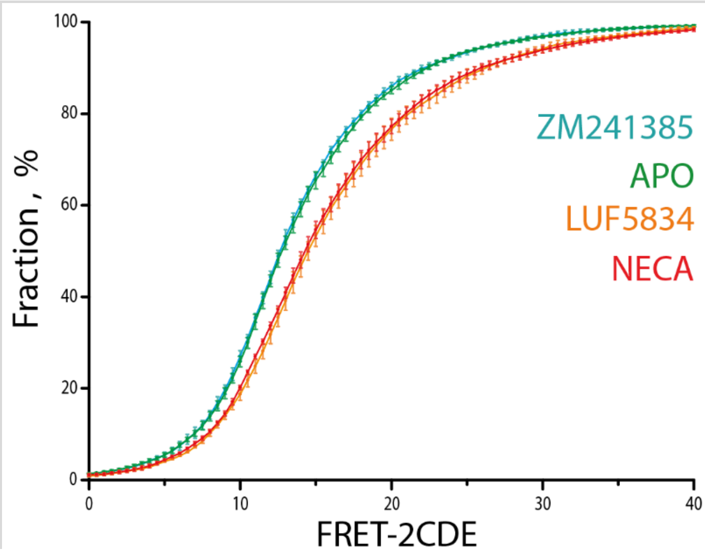

The fraction of molecules with the FRET-2CDE score lower than a threshold is plotted against the threshold value. Mean ± SD between three technical replicas with different protein aliquots are shown for each condition.

Supplementary figure 7. Microtime patterns (top) and filter functions (bottom) used for fFCS.

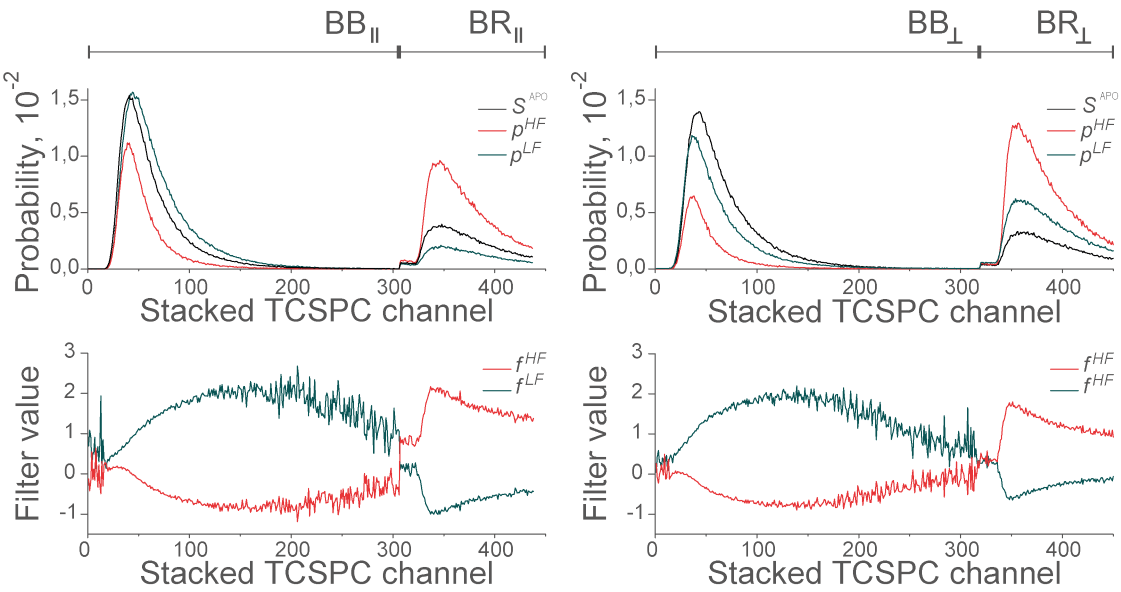

TCSPC patterns for excitation and emission channels $BB_{\parallel}$ and $BR_{\parallel}$ are stacked in the left panels; $BB_{\perp}$ and $BR_{\perp}$ are stacked in the right panels. S^{APO} (black lines) is the microtime-distribution of photons accumulated from the double-labeled apo-A_{2A}AR molecules. p^{LF} (red, top pannel) and p^{HF} (dark green, top panel) show TCSPC-distributions accumulated from bursts with low ($E < 0.3$) and high ($E > 0.7$) burst-wise FRET efficiency (all ligand-bound and apo-conditions merged). f^{LF} and f^{HF} show filter functions used for the 'low-FRET' and 'high-FRET' channels in fFCS with the apo-A_{2A}AR.

Supplementary figure 8. FCS in single-labeled $A_{2A}AR$ molecules reveals photoblinking on a microsecond timescale

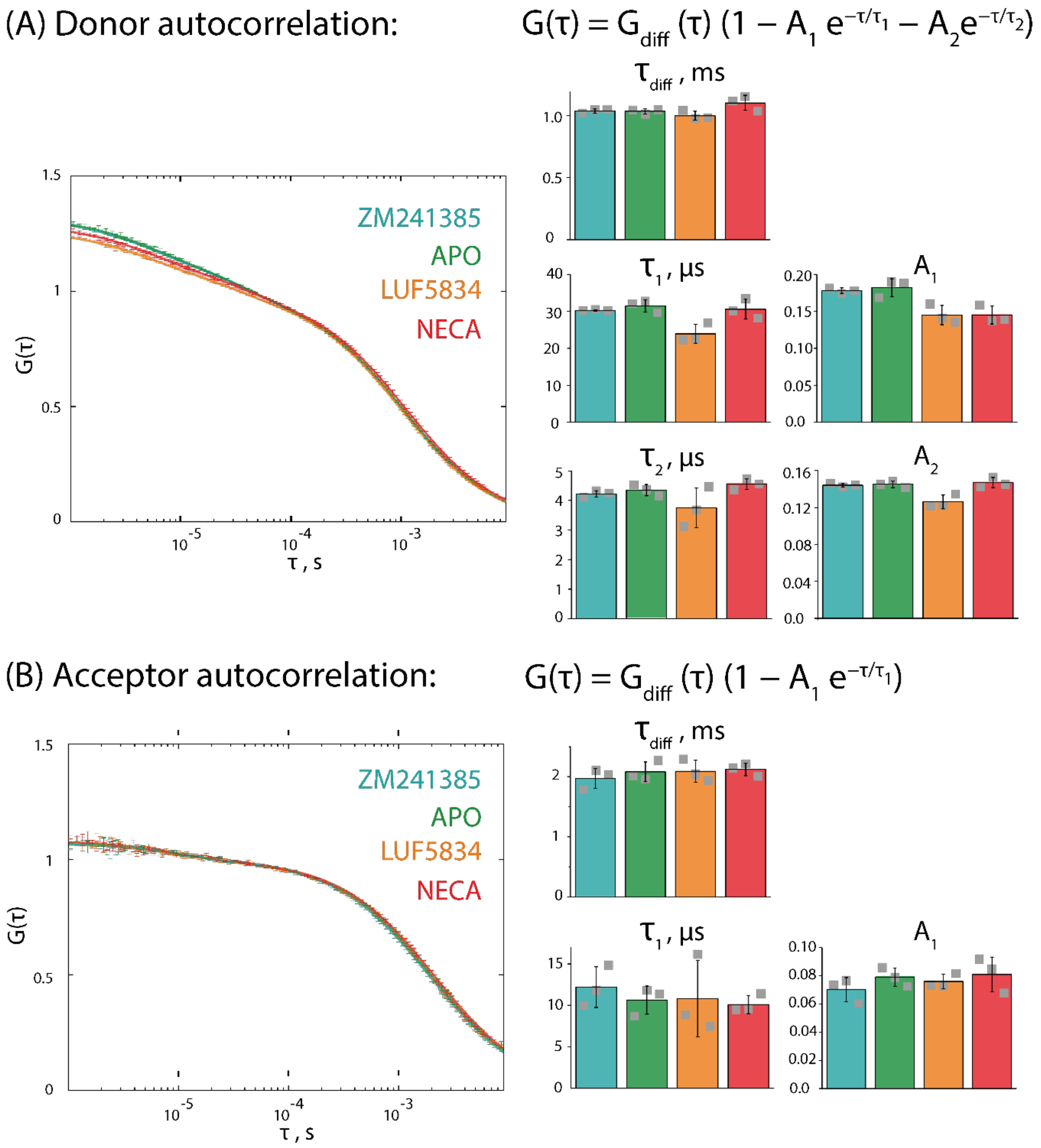

FCS curves were calculated for fluorescence intensity in single-labeled $A_{2A}AR$ molecules. To reduce the bias related to burst selection, a 10 ms time-widow was used. The color-coded bar charts show fitting parameters (mean ± SD for three technical replicas with different protein aliquots; individual data points shown as grey squares).

Supplementary figure 9. Fitting of experimental data for $A_{2A}AR$ with a fFCS model including one anticorrelation term.

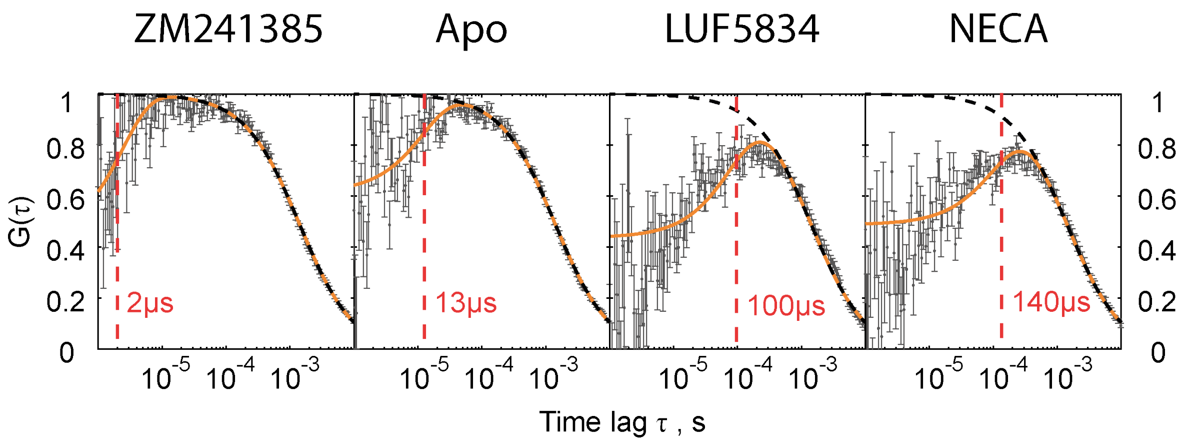

The cross-correlation fFCS function is plotted against the time lag. Experimental points with error bars are shown in gray; the error bars are SDs obtained after splitting the photon data into 10 equally sized bins and correlating each individually. The fitting curves are shown in orange; the diffusion-related terms are shown as dashed black lines; the exchange times derived from the fit are highlighted with vertical red lines. χ_{red}^2 of the global fit is 1.5.

Supplementary figure 10. PDA histograms for the model with two dynamic states

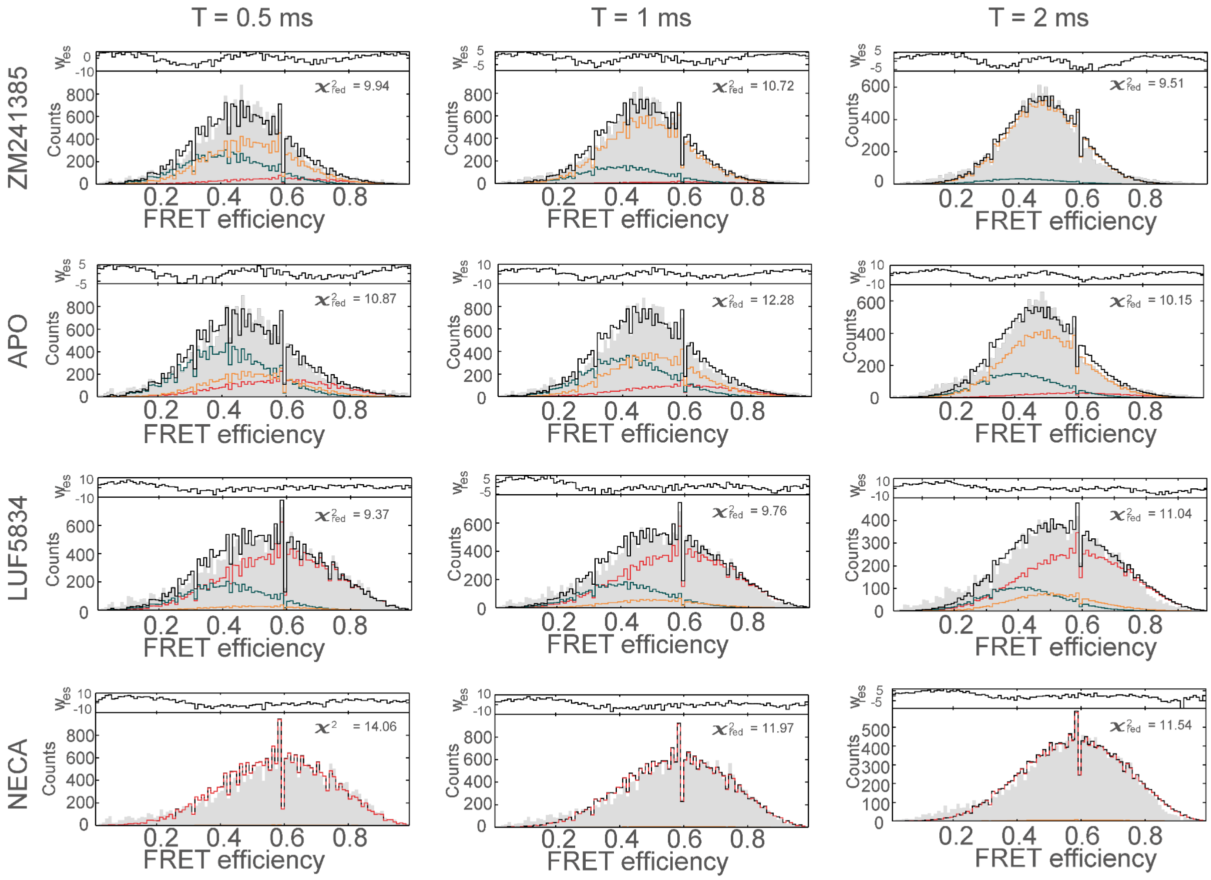

PDA histograms were fit with a sum of two states, allowing the low-FRET and high-FRET states to interconvert. The resulting fit (black line) is a sum of distributions simulated for molecules that stay in the low-FRET (dark cyan line), or high-FRET (red line) state during the entire simulated time-bin, and the distribution for molecules that sample both low-FRET and high-FRET states within the time-bin (orange line). The exchange time was set as a free fit parameter. Columns show PDA distributions for three different time-bin lengths (*T*), rows correspond to A_{2A}AR in different ligand-bound or apo conditions. The experimental distributions are shown as grey bars. The fitting residuals are given on the top of each panel. For the global fit, χ^2_{red} = 10.3.

Supplementary figure 11. PDA histograms for the model with three static states.

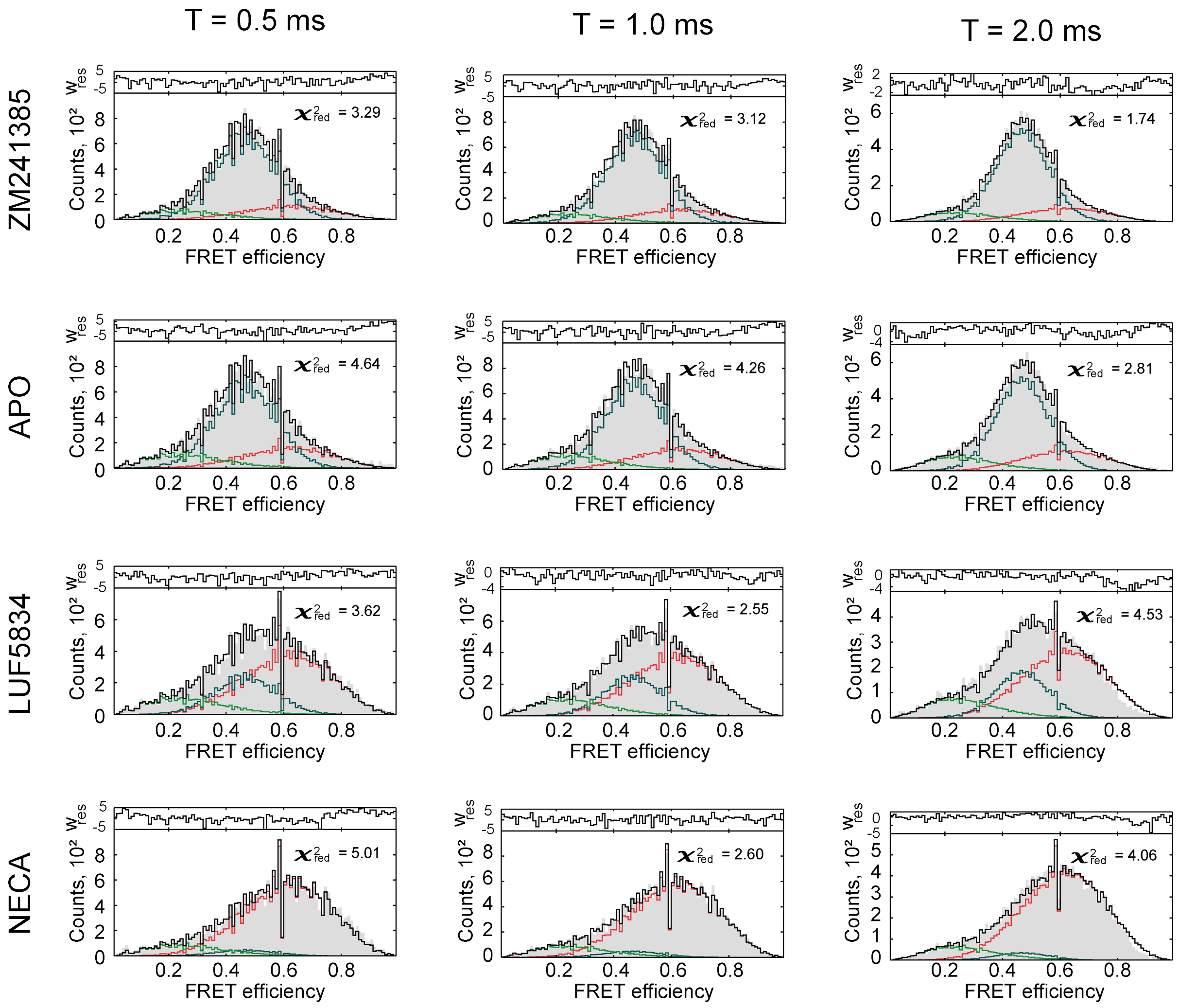

PDA histograms were fit with a sum of three non-interconverting ('static') states. The fitting curve (black line) is shown on top of the experimental distributions (grey bars). Simulated distributions for individual states are shown in light green (low-FRET), dark cyan (medium-FRET), and red (high-FRET) lines. Columns show PDA distributions for three different time-bin lengths (*T*); rows correspond to $A_{2A}AR$ in different ligand-bound or apo conditions. The fitting residuals are given on the top of each panel. For the global fit, χ^2_{red} = 3.2.

Supplementary figure 12. PDA histograms for the fFCS-constrained PDA model.

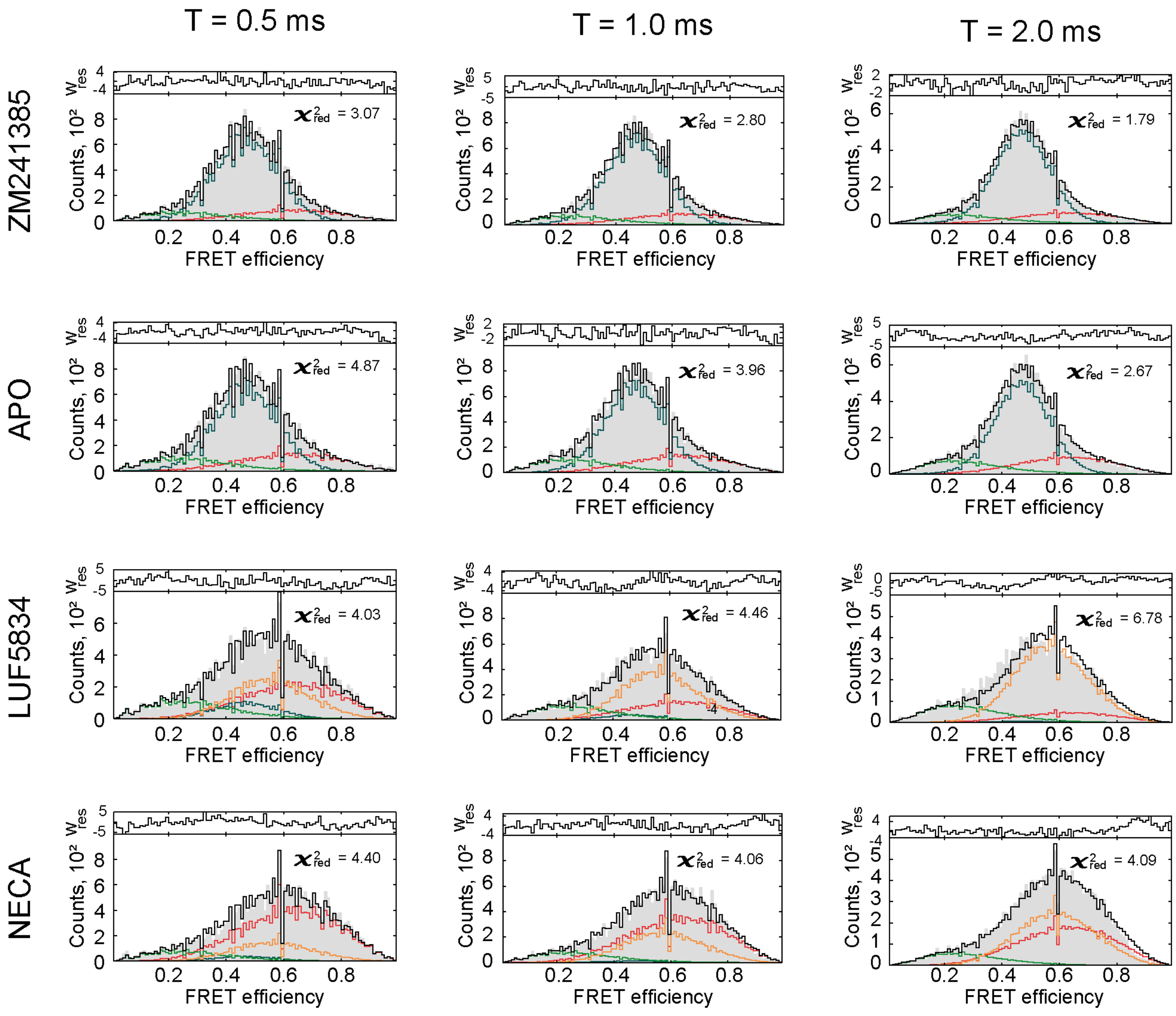

PDA histograms were fit with a sum of three states, allowing the medium-FRET and high-FRET states to interconvert. The longer exchange time derived from fFCS ($\tau_2 =$ 390±80 μs) was used for LUF5834 and NECA. The infinitesimally long exchange times (~100 ms) were used for ZM241385 and apo condition. The resulting fit (black line) is a sum of distributions simulated for molecules that stay in the low-FRET (light green line), medium-FRET (dark cyan line), or high-FRET (red line) state during the entire simulated time-bin, and the distribution for molecules that sample both medium-FRET and high-FRET states within a time-bin (orange line). Columns show PDA distributions for three different time-bin lengths (*T*), rows correspond to $A_{2A}AR$ in different ligand-bound or apo-conditions. The experimental distributions are shown as grey bars. The fitting residuals are given on the top of each panel. For the global fit, χ^2_{red} = 3.6.

Supplementary figure 13. Fluorescence depolarization shows moderate reorientation freedom of the dyes attached to A2AAR in apo and all ligand-bound conditions

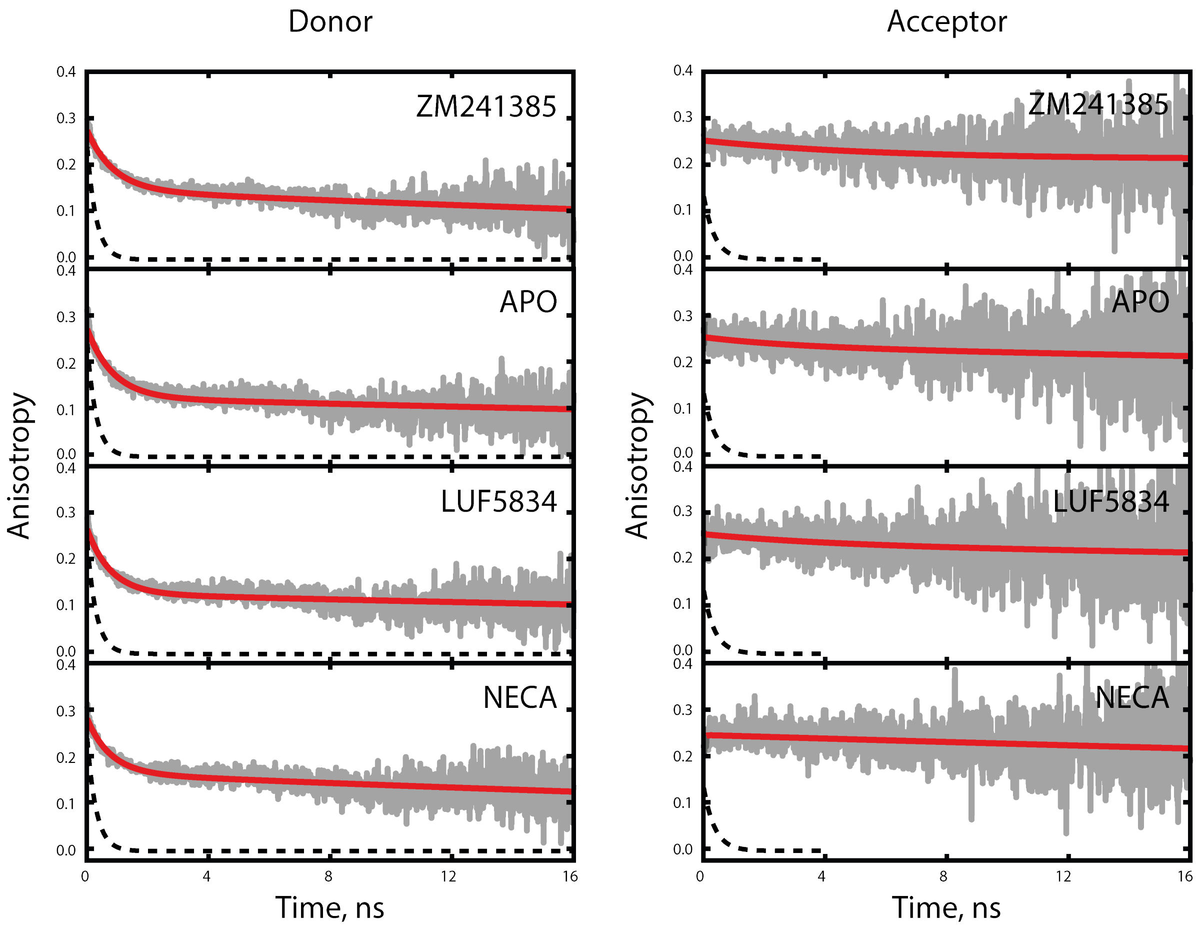

Photons from bursts of only single-labeled A2AAR molecules were analyzed. Experimental data and fit line are shown in grey and in red, respectively. Fluorescence depolarization curves for a free dye are depicted with dashed black lines.

Supplementary figure 14. MD simulations suggest that a dye attached to TM6 can enter the G-protein-binding cavity upon $A_{2A}AR$ activation.

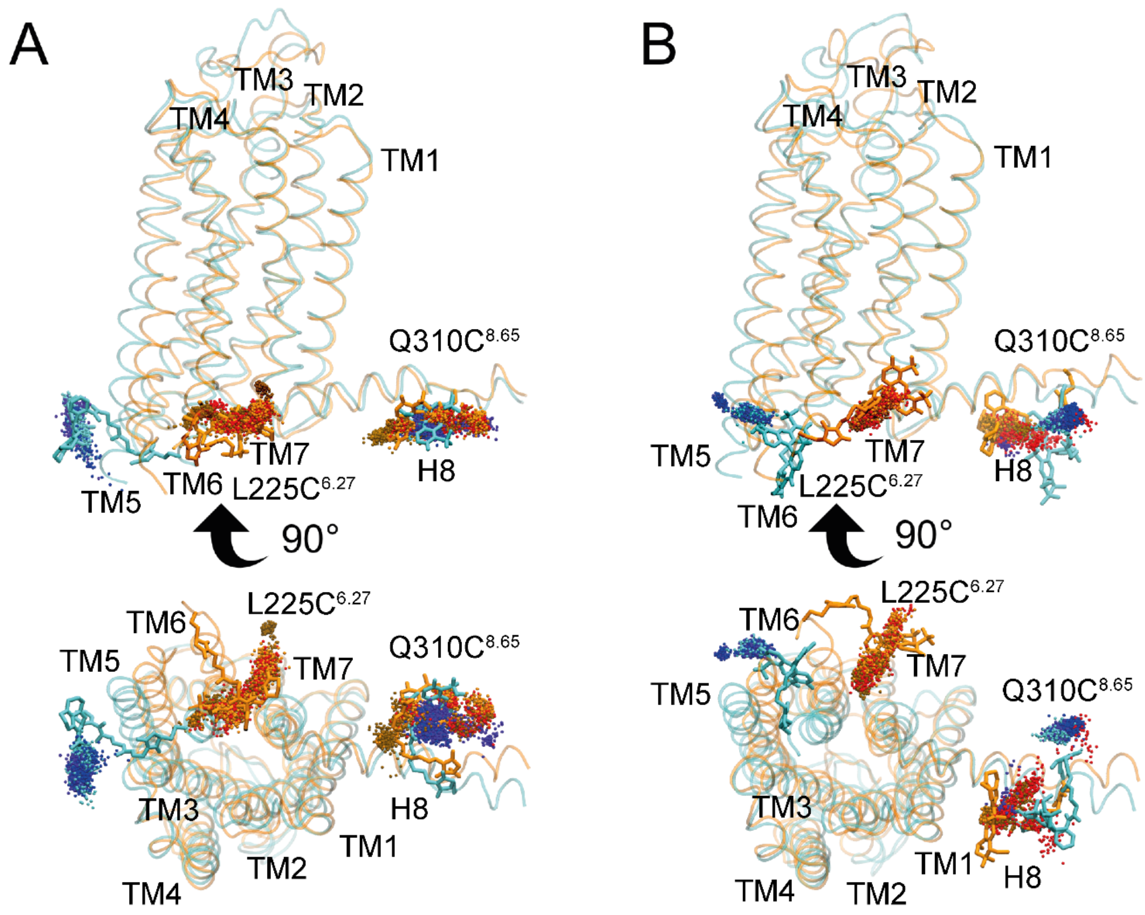

The inactive (based on PDB: 3PWH) and fully active (targeted to PDB: 5G53) structures of $A_{2A}AR$ after equilibration are shown in cyan and orange, respectively. Representative positions of the dyes are shown with sticks of similar colors for inactive and active structures, respectively. Dots indicate the geometrical centers of the fluorophores sampled in 1-μs-long MD trajectories with 1-ns time-steps for each $A_{2A}AR$ state and in each of three MD replicas (three active replicas are shown in orange, red, and ochre; three inactive replicas – in cyan, blue, and violet); in panel A, Atto647N is attached to $L225C^{6.27}$, Alexa488 is attached to $Q310C^{8.65}$. In panel B, the labels are swapped, i.e., Alexa488 is attached to $L225C^{6.27}$, Atto647N is attached to $Q310C^{8.65}$. Regardless of the specific fluorophore type, the dye attached to TM6 enters the G-protein-binding cavity between TM3, TM5, TM6, and TM7 in the active $A_{2A}AR$ structure.

Supplementary figure 15. Inter-label distances calculated from MD simulations.

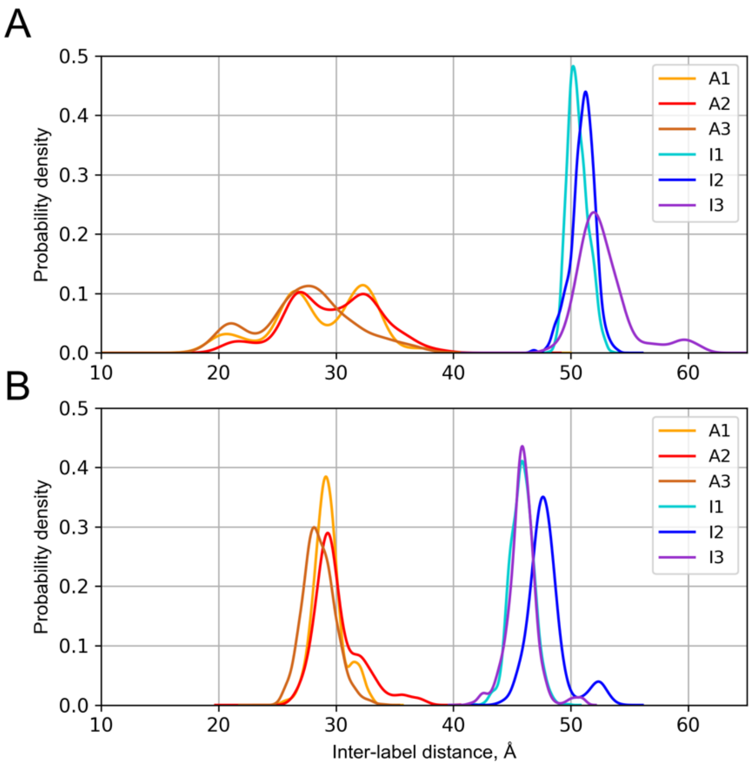

Inter-label distances calculated from triplicate MD simulations (I1-3 correspond to simulations of the inactive (based on PDB: 3PWH) protein; A1-3 – the fully active (targeted to PDB: 5G53) protein). In panel A, Atto647N is attached to L225C$^{6.27}$, Alexa488 is attached to Q310C$^{8.65}$; in panel B, the labels are swapped, i.e., Alexa488 is attached to L225C$^{6.27}$, Atto647N is attached to Q310C$^{8.65}$. Color code matches that in Figure S14, i.e., three active replicas are shown in orange, red, and ochre; three inactive replicas – in cyan, blue, and violet.

Supplementary figure 16. Convergence of MD sampling

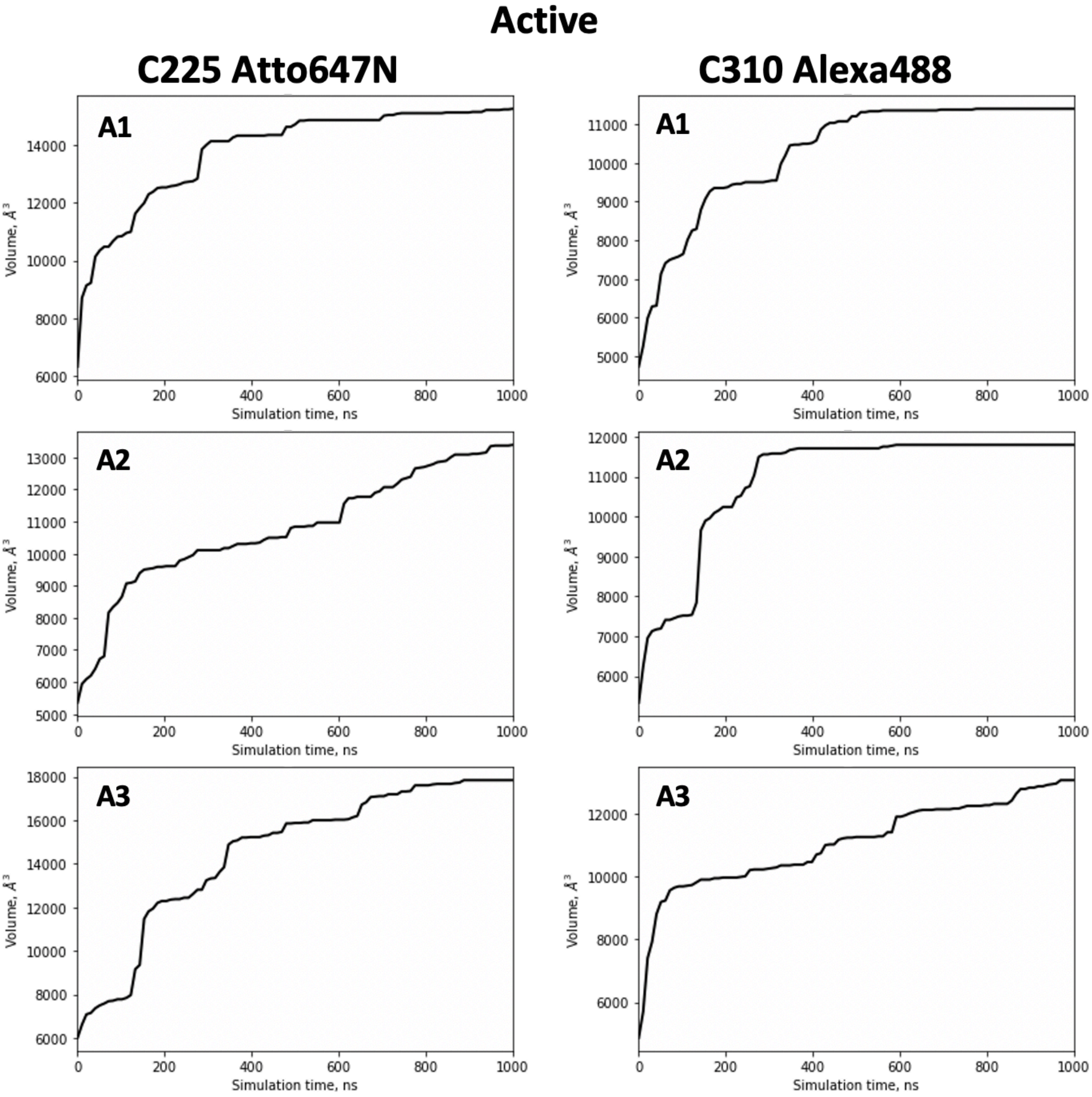

Inactive

C225 Atto647N **C310 Alexa488**

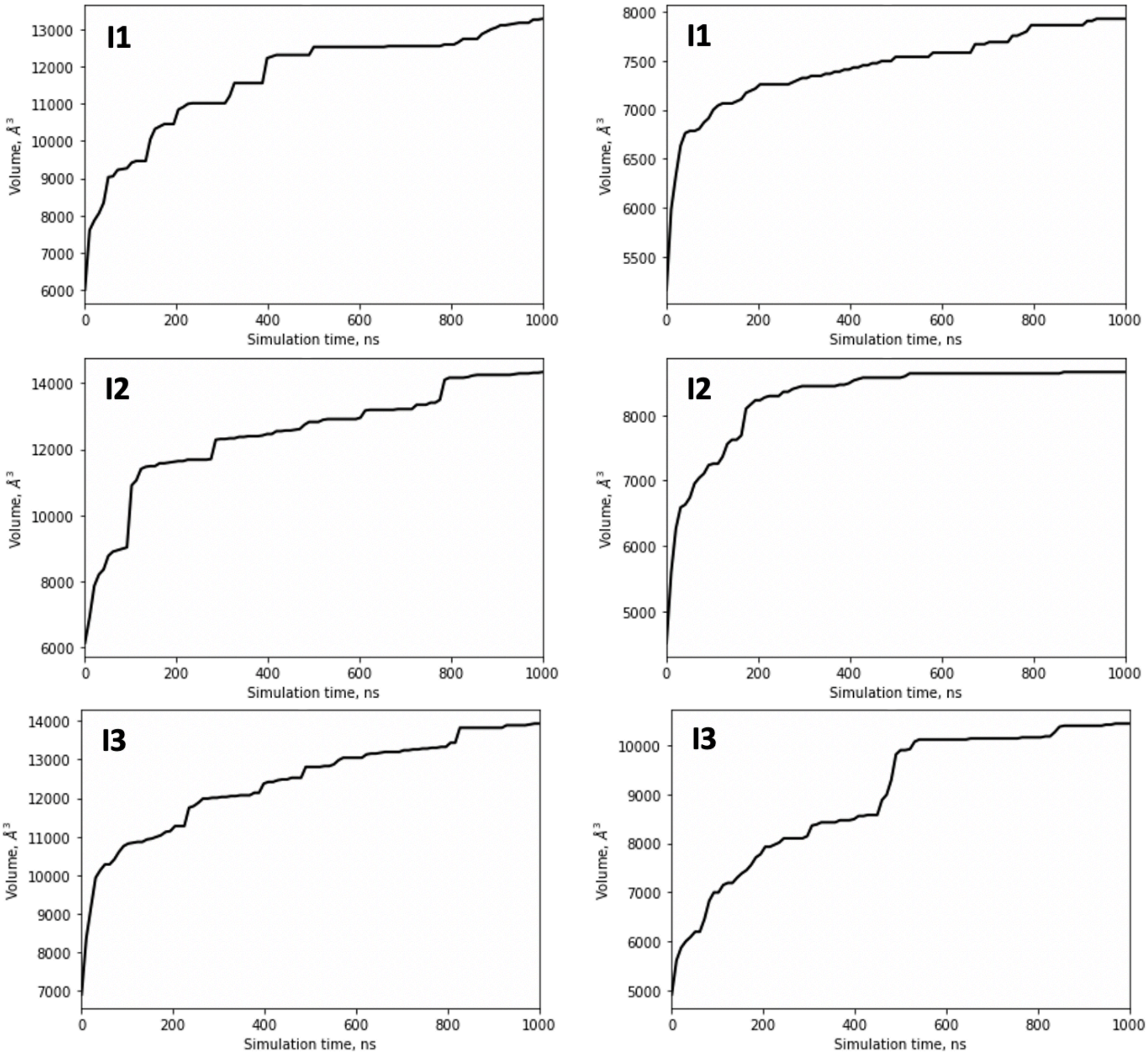

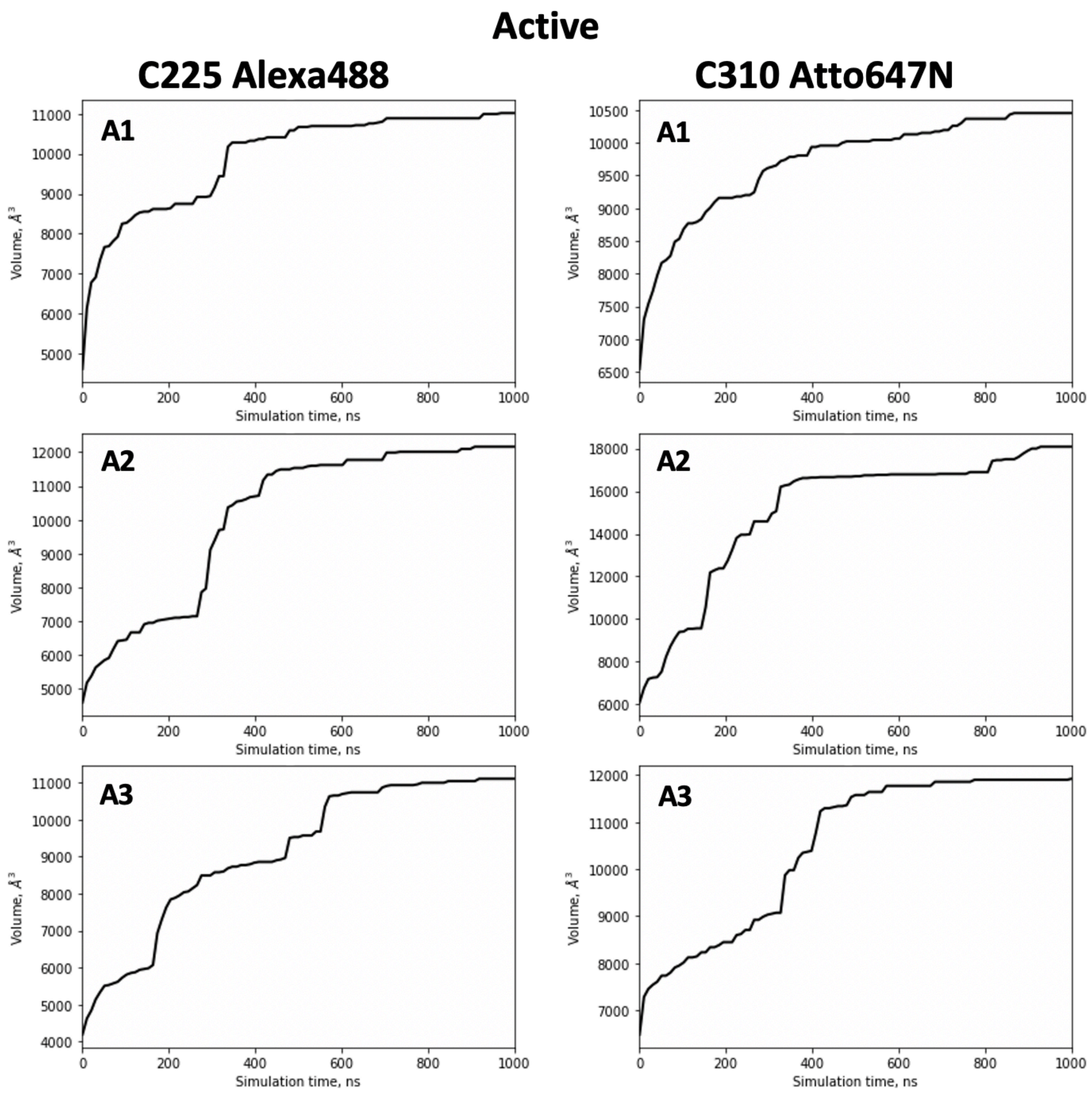
Active
C225 Alexa488
C310 Atto647N
A1
A2
A3
Volume, Å³
Simulation time, ns

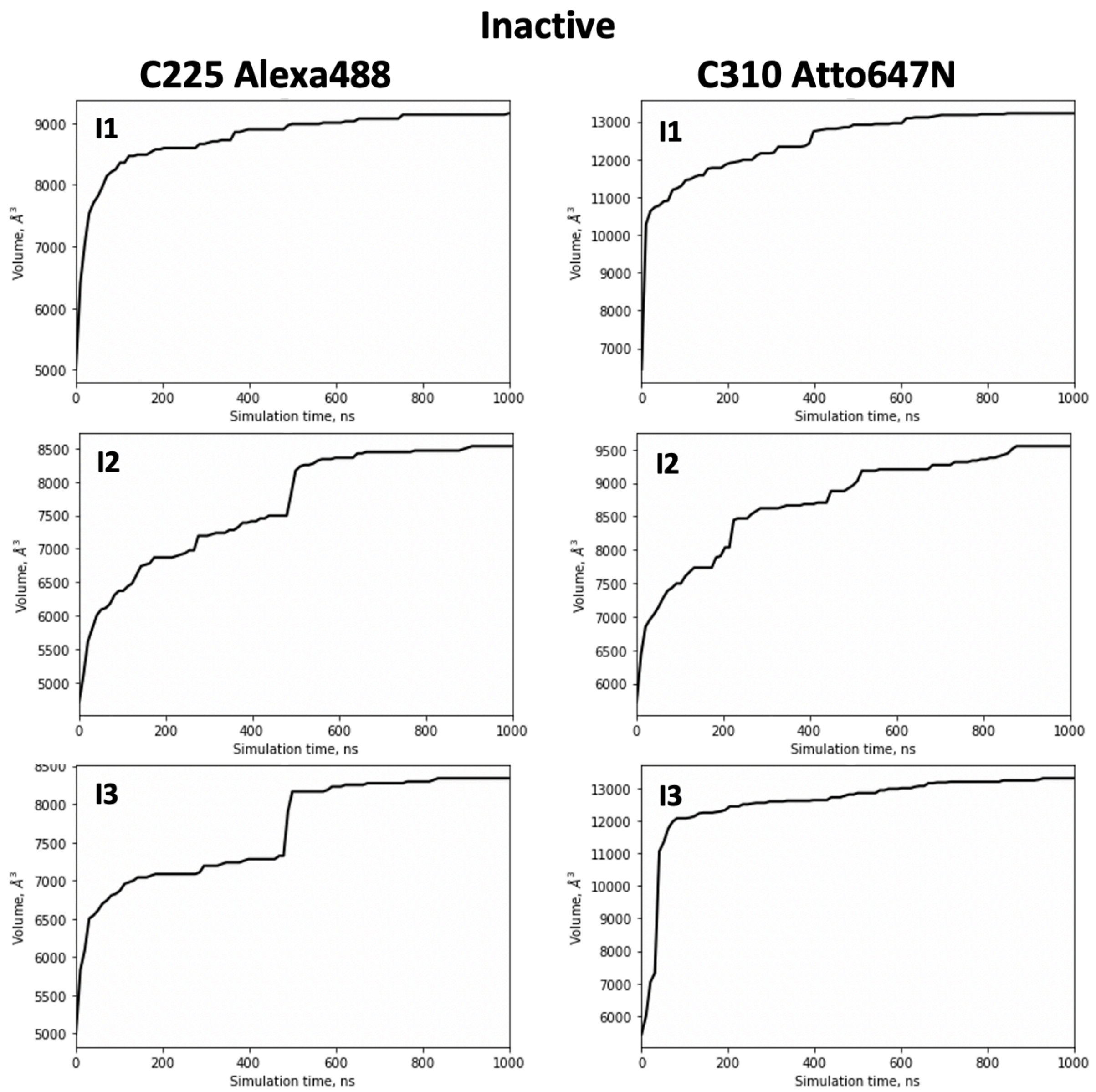

Cumulative volume available for fluorescent labels in course of molecular dynamics simulations was used to evaluate the convergence of MD trajectories. Each plot shows the evolution of the total available volume as a function of simulation time for each label in two alternative labeling schemes (C225 Alexa488, C310 Atto647N, and C225 Atto647N and C310 Alexa488). The results of analysis for triplicate simulations conducted for the active and inactive protein conformations are shown (A1-3, and I1-3).

Supplementary figure 17. Distributions of the burst-wise fluorescence lifetime (τ) and anisotropy (r) for fluorophores in (A) donor-only and (B) acceptor-only A_{2A}AR populations.

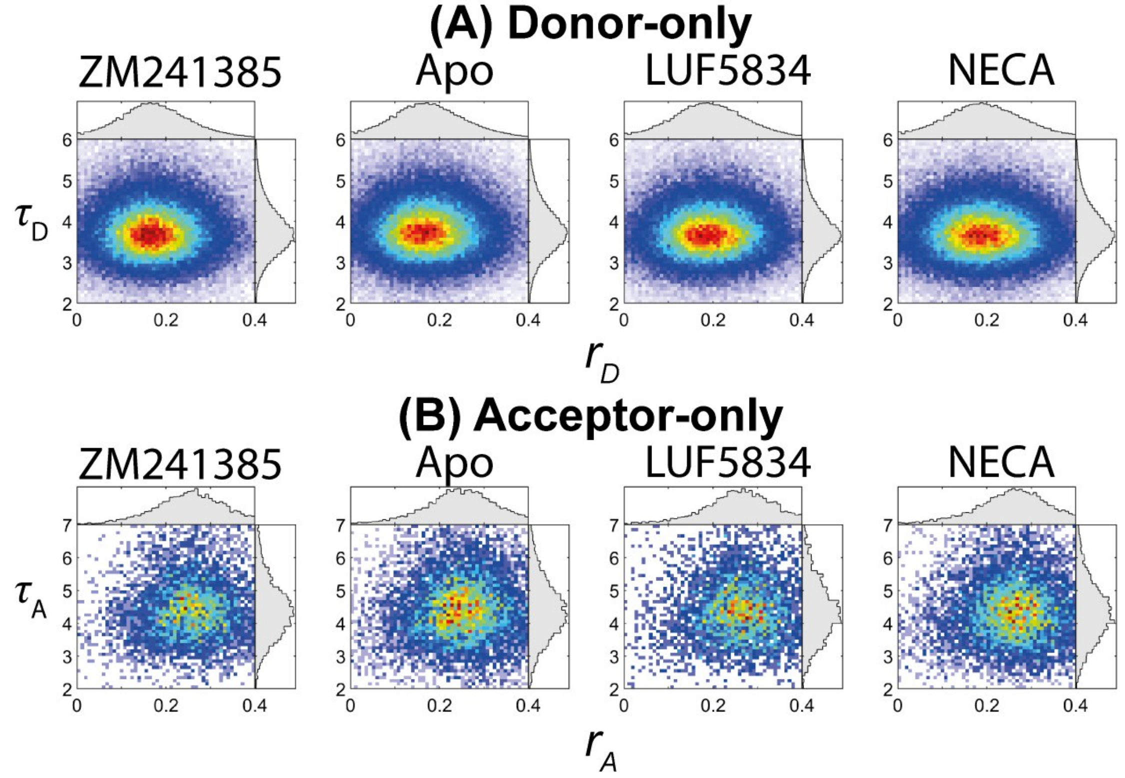

Supplementary table 1. Labeling efficiencies

Mutations	Alexa488, %	Atto643, %
$Q310C^{8.65}/L225C^{6.27}$	26	8
WT	1.1	0.4

Labeling efficiencies (in %) of the wild type and double-cysteine mutant of $A_{2A}AR$ with Alexa488 and Atto643 dyes based on the corresponding absorption, assuming one binding site for each dye on a single protein.

Supplementary table 2. Mean burst-wise parameters

	ZM241385	APO	LUF5834	NECA
r_D	0.173±0.001	0.173±0.002	0.191±0.004	0.190±0.004
τ_D	3.77±0.02	3.80±0.02	3.75±0.01	3.74±0.01
$r_{D(A)}$	0.195±0.002	0.202±0.002	0.211±0.002	0.213±0.006
$\tau_{D(A)}$	2.50±0.06	2.50±0.04	2.42±0.11	2.20±0.02
E	0.45±0.01	0.45±0.01	0.50±0.02	0.55±0.01
$r_{A(D)}$	0.261±0.002	0.253±0.004	0.260±0.001	0.261±0.002
$\tau_{A(D)}$	4.57±0.02	4.55±0.08	4.56±0.02	4.53±0.01
FRET-2CDE	13.8±0.2	14.0±0.2	16.1±0.5	15.8±0.3
r_A	0.261±0.003	0.252±0.004	0.265±0.002	0.263±0.001
τ_A	4.51±0.04	4.47±0.11	4.45±0.05	4.43±0.02

Mean burst-wise parameters are given with SD between the mean parameter values measured in three technical replicas with different protein aliquots. Donor and acceptor fluorescence lifetime (τ_D, τ_A) and anisotropy (r_D, r_A) were determined for single-labeled molecules. For double-labeled molecules, donor fluorescence lifetime ($\tau_{D(A)}$) and anisotropy ($r_{D(A)}$), FRET efficiency (E), acceptor fluorescence lifetime ($\tau_{A(D)}$) and acceptor anisotropy ($r_{A(D}$) were determined.

Supplementary table 3. Fitting parameters for fFCS cross-correlation curves.

(A)

	τ_{diff}, ms	A	τ, µs
ZM241385	1.44±0.05	0.6±0.3	2.4±1.5
APO	1.44±0.05	0.39±0.08	13±4
LUF5834	1.44±0.05	0.56±0.04	100±20
NECA	1.44±0.05	0.51±0.04	140±30

(B)

	τ_{diff}, ms	A_1	A_2	τ_1, µs	τ_2, µs
ZM241385	1.38±0.04	0.5±0.2	-	3±1	-
APO	1.38±0.04	0.38±0.06	-	14±4	-
LUF5834	1.38±0.04	0.54±0.05	0.36±0.04	18±4	390±80
NECA	1.38±0.04	0.38±0.08	0.37±0.04	16±6	390±80

(A) Fitting parameters of fFCS cross-correlation curves with one anticorrelation term for apo and ligand-bound conditions. τ_{diff} was optimized globally across all four datasets. χ_{red}^2 = 1.5 **(B)** Fitting parameters of fFCS cross-correlation curves with one and two anticorrelation terms for the apo/antagonist-bound and the agonist-bound receptor, respectively; τ_{diff} and τ_2 were optimized globally across all four datasets. χ_{red}^2 = 1.1. The half-widths of the 95% confidence intervals are given as fitting errors.

Supplementary table 4. Fitting parameters for a PDA model with two dynamic states.

(A)

	R, Å	σ, Å
MF*	52.0	3.5
HF*	46.5	4.8

(B)

	τ_{ex} , ms	LF*, %	HF*, %
ZM241385	0.3	67	33
Apo	0.7	64	36
LUF5834	4.6	27	73
NECA	0.06	<0.1	>99.9

Fitting PDA parameters for a model with two interconverting states with a free exchange time. **(A)** The means (*R*) and standard deviations (σ) of the Gaussian apparent distance distributions for individual states (low-FRET – LF*, high FRET –HF*). **(B)** Populations of the three states as well as the optimal exchange times (columns) for the ligand-bound or apo-A_{2A}AR (rows).

Supplementary table 5. Fitting parameters for a PDA model with three static states.

(A)

	R, Å	σ, Å
LF	58.3	5.3
MF	50.1	2.0
HF	45.4	4.1

(B)

	LF, %	MF, %	HF, %
ZM241385	10	72	11
APO	15	63	22
LUF5834	17	25	58
NECA	12	4	83

(A) The means (*R*) and standard deviations (σ) of the Gaussian apparent distance distributions for individual states (low-FRET - LF, medium FRET - MF, high FRET -HF). **(B)** Populations of the three states (columns) for the ligand-bound or apo-A_{2A}AR (rows).

Supplementary table 6. Fitting parameters for an fFCS-constrained PDA model.

(A)

	R, Å	σ, Å
LF	57.9	6.0
MF	50.0	2.1
HF	45.1	4.9

(B)

	τ_{ex}, ms	LF, %	MF, %	HF, %
ZM241385	> 2	11±3	74±3	15±1
APO	> 2	15±5	64±6	20±3
LUF5834	0.39	19±6	24±8	56±8
NECA	0.39	11±4	12±1	76±3

(A) The means (*R*) and standard deviations (σ) of the Gaussian apparent distance distributions for individual states (low-FRET – LF, medium FRET – MF, high FRET – HF). **(B)** Populations (mean ± SD of three technical replicas with different protein aliquotes) of the three states as well as their exchange times (columns) for the ligand-bound or apo-A_{2A}AR (rows).

Supplementary table 7. Fitting parameters for ensemble-based fluorescence depolarization curves

(A) Donor depolarization

	ρ_F, ns	r_0	r_p
ZM241385	1.1±0.4	0.269±0.013	0.14±0.01
APO	1.0±0.3	0.265±0.010	0.12±0.02
LUF5834	0.9±0.3	0.262±0.006	0.12±0.01
NECA	0.9±0.3	0.278±0.011	0.16±0.01

(B) Acceptor depolarization

	ρ_F, ns	r_0	r_p
ZM241385	4±2	0.253±0.003	0.22±0.01
APO	3±2	0.256±0.001	0.23±0.02
LUF5834	5±2	0.254±0.005	0.22±0.01
NECA	3±2	0.252±0.010	0.23±0.02

Mean ± SD between three technical replicas with different protein aliquots are given.

Supplementary References

1. Vandenberk, N., Barth, A., Borrenberghs, D., Hofkens, J. & Hendrix, J. Evaluation of Blue and Far-Red Dye Pairs in Single-Molecule Förster Resonance Energy Transfer Experiments. *J. Phys. Chem. B* **122**, 4249–4266 (2018).
