# Supplementary material for: Sub-millisecond conformational dynamics of the A2A adenosine receptor revealed by single-molecule FRET
Source: Commun Biol. 2023 Apr 3;6:362. doi: 10.1038/s42003-023-04727-z (PMC10070357; doi:10.1038/s42003-023-04727-z)
Supplement: Supplementary file 3 — Description of Additional Supplementary Data [file 42003_2023_4727_MOESM3_ESM.docx]

**Description of Additional Supplementary Files**

**File name:** Supplementary Data 1

**Description:** The source data for Figure 2H.

**File name:** Supplementary Data 2

**Description:** The source data for box plots in Figure 3A.

**File name:** Supplementary Data 3

**Description:** Topology for Atto647Nmaleimide.

**File name:** Supplementary Data 4

**Description:** Topology for Alexa488- C5-maleimide.
